# The Wisdom in Teeth: Neuronal Differentiation of Dental Pulp Cells

**DOI:** 10.1089/cell.2022.0102

**Published:** 2023-02-14

**Authors:** Bendegúz Sramkó, Anna Földes, Kristóf Kádár, Gábor Varga, Ákos Zsembery, Karolina Pircs

**Affiliations:** ^1^ HCEMM-SU Neurobiology and Neurodegenerative Diseases Research Group, Budapest, Hungary.; ^2^ Institute of Translational Medicine, Semmelweis University, Budapest, Hungary.; ^3^ Department of Oral Biology, Faculty of Dentistry, Semmelweis University, Budapest, Hungary.; ^4^ Laboratory of Molecular Neurogenetics, Department of Experimental Medical Science, Wallenberg Neuroscience Center and Lund Stem Cell Center, Lund University, Lund, Sweden.

**Keywords:** dental pulp stem cells, neural differentiation, mesenchymal stem cells, reprogramming, neural crest

## Abstract

Mesenchymal stem/stromal cells (MSCs) are found in almost all postnatal organs. Under appropriate environmental cues, multipotency enables MSCs to serve as progenitors for several lineage-specific, differentiated cell types. *In vitro* expansion and differentiation of MSCs give the opportunity to obtain hardly available somatic cells, such as neurons. The neurogenic potential of MSCs makes them a promising, autologous source to restore damaged tissue and as such, they have received much attention in the field of regenerative medicine. Several stem cell pool candidates have been studied thus far, but only a few of them showed neurogenic differentiation potential. Due to their embryonic ontology, stem cells residing in the stroma of the dental pulp chamber are an exciting source for *in vitro* neural cell differentiation. In this study, we review the key properties of dental pulp stem cells (DPSCs), with a particular focus on their neurogenic potential. Moreover, we summarize the various presently available methods used for neural differentiation of human DPSCs also emphasizing the difficulties in reproducibly high production of such cells. We postulate that because DPSCs are stem cells with very close ontology to neurogenic lineages, they may serve as excellent targets for neuronal differentiation *in vitro* and even for direct reprogramming.

## Introduction

Cell fate determination is a general process in differentiated living organisms (Furlong, 2010). Since cellular differentiation leads to specialized, functionally active cells, understanding and influencing these processes can potentially help restore tissue integrity and functionality (Ratcliffe et al, 2013).

During embryonic development, the derivatives of the fertilized oocyte are differentiated into specified cells while losing their potential for producing other cell types (Bertrand and Hobert, 2010). Stem cells, the immature precursors of specialized cells, are able to self-renew and differentiate into several lineages (Kolios and Moodley, 2013). Embryonic stem cells (ESCs) were the first that were differentiated into specific lineages, although their availability is limited due to biological and ethical issues (Charitos et al, 2021; Martello and Smith, 2014). Later on, the discovery and widespread usage of patient-derived induced pluripotent stem cells (iPSCs) retrieved from adult human tissues completely revolutionized the reprogramming field by allowing the use of different cell types such as blood cells and fibroblasts as cell sources and thus avoiding ethical issues (Andrews, 2021).

As a result, several novel cellular reprogramming techniques have emerged such as direct reprogramming or so-called transdifferentiation of fibroblasts into other cell types, including neurons (Bocchi et al, 2022; Grath and Dai, 2019; Vierbuchen et al, 2010; Wapinski et al, 2013). Direct conversion provides access to donor-derived, *in vitro* reprogrammed cells, which, in contrast to iPSCs, are nonclonal, and do not undergo a PSC or progenitor stage at any point during the conversion, therefore preserving the epigenetic aging signatures of the donor cells (Drouin-Ouellet et al, 2017b; Frobel et al, 2014; Kane and Sinclair, 2019; Mertens et al, 2016; Pircs et al, 2022; Yang et al, 2015).

Similar to iPSCs, the most commonly used cell pool for direct conversion up to date are fibroblasts, almost terminally differentiated somatic cells. The embryonic ontology of cells is the key aspect of tissue repair and engineering. Consequently, the use of fibroblasts as a source for differentiation and reprogramming is challenging (Kolios and Moodley, 2013; Perczel-Kovach et al, 2021; Shi et al, 2001).

Mesenchymal stem/stromal cells (MSCs) are multipotent cells found in almost all postnatal organs and represent an attractive cell pool for reprogramming methods. Their multipotency makes it possible after extraction and under appropriate environmental cues, to differentiate them into germ line-specific lineages (Pittenger et al, 2019). Various types of MSCs have been described according to their adherence to plastic, specific surface antigen expression, and multipotency (Dominici et al, 2006). MSCs derived from bone marrow (BM) and Wharton jelly are widely used for research and therapeutic purposes (Hong, 2022; Liau et al, 2020). MSCs originating from other tissues, including the dental pulp isolated from third molars (wisdom teeth), have also been studied and used.

In this review, we describe the origin and cellular specification of the ectomesenchymal human dental pulp stem cells (hDPSCs). We summarize how these cells were previously differentiated *in vitro* to a neuronal fate. All protocols used allogenic cell sources and presented neurogenic differentiation of hDPSCs. The reviewed differentiation protocols demonstrate indirect ways to get insight into the epigenetic landscape of hDPSCs. Lastly, we propose an exciting alternative method, direct reprogramming of hDPSCs for neuronal differentiation in the future.

## Dental Pulp Stem Cells

The dental pulp is a soft tissue, located in the pulp chamber of the teeth. Most importantly, it comprises dentin producing odontoblasts, fibroblast-like cells, neural fibers, and blood vessels. Similar to other stromal organs, the stroma of the dental pulp and their neighboring tissues contains stem cells with MSC properties. These dental-derived cell pools include stem cells from human exfoliated deciduous teeth, stem cells from apical papilla, periodontal ligament stem cells, and also DPSCs, which are able to differentiate into several lineages showing similar embryonic ontology, differentiation potency, and cellular morphology.

**FIG. 1.**
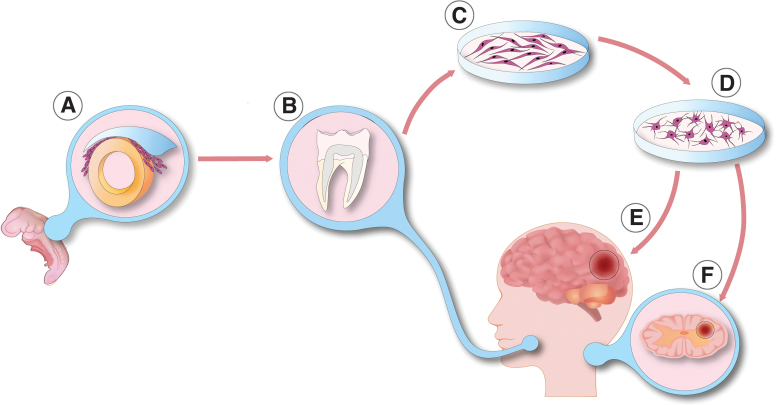
Neuronal differentiation of dental pulp cells. **(A)** During the fourth week of embryonic life—within the ectoderm—dorsally from the neural tube, neural crest cells start to migrate into distinct parts of the developing organs. Later, derivatives of neural crest cells take part in the formation of different craniofacial organs, including the dental pulp complex. **(B)** Stromal cells from the dental pulp are an easily obtainable source of cells extracted from deciduous or permanent tooth dental. **(C)** Dental pulp stem cells are spindle-shaped, multipotent stem cells, residing in the stroma of the pulp chamber. After isolation, dental pulp stem cells can start to proliferate in optimal culture conditions. **(D)** These cells are multipotent and in accordance with their embryonic ontology, under appropriate environmental circumstances, dental pulp stem cells can differentiate into functionally active neuronal cells. **(E, F)** These *in vitro* differentiated neuronal cells provide a promising alternative cell source for central nervous system regeneration after ischemic brain damage **(E)** or spinal cord injury **(F)**.

In this review, we focus on hDPSCs, which are the most widely used and understood dental-derived stem cell type (Bansal and Jain, 2015; Campanella, 2018; Marrelli et al, 2015; Mayo et al, 2014; Sharpe, 2016). Human DPSCs are easily harvested neural crest-derived mesenchymal-like stem cells residing in the dental pulp tissue *in vivo*. They have a spindle-shaped morphology and are easily available from impacted third molars (Fig. 1) (Huang et al, 2009). Their biological role is well described. Dentin is formed by odontoblasts, which are terminally differentiated hard-tissue forming cells in the dentin-pulp complex. Postnatal cells in the dental papilla constitute the stem cell pool called DPSCs, which differentiate into odontoblasts. Therefore, DPSCs play a pivotal role in dentinogenesis (Gronthos et al, 2000). DPSCs are an ideal source of MSCs *in vitro*, due to their high proliferation rate, self-renewal capability, and easy accessibility.

According to the cross-lineage boundaries, hDPSCs may provide a potential cell pool for regenerative medicine and clinical tissue engineering (Fang et al, 2013; Naz et al, 2022). In line with this, many of the hDPSC-based researches demonstrate their beneficial effect in endodontic therapies (Nakashima et al, 2022). Moreover, hDPSCs promote *in vivo* tissue regeneration after transplantation into animal models with pathologies associated with cardiological, neurological, and ophthalmological disorders (Anitua et al, 2018; Fang et al, 2013; Shi et al, 2020). Focusing on neurological injuries, Yang et al (2017) transplanted different types of dental stem cells (DSCs), including hDPSCs, into transected rat spinal cord and observed pure functional recovery after 8 weeks. They hypothesized that DSCs, due to inhibition of interleukin-1beta, reduce inflammation in the spinal cord of the rats. DSCs also inhibited injury-induced neuronal, astrocytic, and oligodendrocytic apoptosis.

Multiple inhibitions of axon growth inhibitors through paracrine mechanisms were also described. Interestingly after spinal cord injury, DSCs were able to differentiate into mature neurons and oligodendrocytes in the damaged tissue. This phenomenon has also been reported by others (Sakai et al, 2012; Yamamoto et al, 2014; Yang et al, 2017).

Although several methods were developed to isolate hDPSCs from the dental pulp, isolated hDPSCs—similarly to other MSCs—invariably comprise many various subpopulations according to their biological and regenerative characteristics (Gronthos et al, 2002). These traits present a major obstacle to the regenerative utilization of hDPSC-based therapies. By including divergent proliferation potentials, differentiation properties to lineage-specific fate determination, and cell surface markers, hDPSCs represent a heterogeneous source of MSCs (Kok et al, 2022). Consequently, one of the main challenges using hDPSCs as a cellular source for routine regenerative therapy is the identification of particular cell surface markers and discrimination of different hDPSC subpopulations in terms of their proliferation and differentiation potential, immunomodulatory effect, and other regenerative properties.

Several studies reported candidate markers of hDPSCs, however, no specific hDPSC marker has been identified thus far (Tatullo et al, 2015). The main MSC markers are CD29, CD44, CD73, CD90, CD105, CD146, CD166, CD271, and STRO-1, which are all widely expressed across hDPSC subpopulations (Gronthos et al, 2002; Perczel-Kovach et al, 2021). These markers exemplify the heterogeneous nature of DPSCs (Kawashima, 2012). According to the neighboring persistence of the perivascular niche, subpopulations of hDPSCs express STRO-1, STRO-3, platelet-derived growth factor-beta, vascular endothelial growth factor 1, and CD146 (Shi and Gronthos, 2003). Several studies reported ESC marker expression in hDPSCs: octamer binding transcription factor 4 (*OCT-4*), homeobox transcription factor nanog, SRY-box transcription factor 2 (*SOX-2*), stage-specific embryo antigen 4 (*SSEA4*), and snail family transcriptional repressor 2 (*Slug*), which influence self-renewal capacity and multipotency (Huang et al, 2009; Kunimatsu et al, 2018).

In addition to the differentiation and proliferation characteristics, subpopulations of hDPSCs can also exhibit anti-inflammatory and immunomodulatory properties (Demircan et al, 2011; Foldes et al, 2016; Racz et al, 2014). Accordingly, hDPSCs induce lymphocyte activation through the secretion of pleiotropic regulators, modulation of antigen-presenting cells, and by building cell-to-cell interactions involved in immune cell adhesion and migration (Ma and Chan, 2016). Several studies reported an increased expression of CD90 on distinct subpopulations of hDPSCs, a commonly used bone marrow-derived MSC marker (Agha-Hosseini et al, 2010; Alongi et al, 2010).

DSCs, including DPSCs, are derived from the neural crest. During embryological development, the central nervous system (CNS) is formed by the ectoderm, the outermost germ layer of the gastrulated embryo (Nikolopoulou et al, 2017). In chordate, CNS precursors are related to the neural tube, which is formed by a certain process called neurulation (Schoenwolf and Smith, 1990). Dorsally from the neural tube during neurulation, certain types of neurectoderm cells disconnect from the epidermis and start to migrate into distinct parts of the developing organism. These migrating cells are the earliest precursors of neural crest cells (Fig. 1). Derivatives of the neural crest are melanocytes, peripheral and enteric neurons, glial cells, different types of connective tissue, and stromal cells mostly in the cardiac and the craniofacial region (Rothstein and Simoes-Costa, 2022; Song et al, 2022). The dental pulp is also formed from neural crest cells.

Progenitors of DPSCs migrate from the cranial neural crest into the pharyngeal arches, and after an ectomesenchymal transition, DPSCs reside in the dental pulp (Ibarretxe et al, 2012; Miletich and Sharpe, 2004). Indeed, derivatives of these cells express both ectodermal and mesodermal markers (Lee et al, 2011). The expression of various neural lineage markers were reported: *CD117* (*c-Kit*), *CD271*, nestin (*NES*), glial fibrillary acidic protein (*GFAP*), beta-III tubulin (*TUBB3*), *S100*, *Notch 2*, Musashi RNA binding protein 1 (*MSI1*), synaptophysin (*SYP*), and microtubule associated protein 2 (*MAP2*) (Karaoz et al, 2011; Kawashima, 2012; Kiraly et al, 2011; Kiraly et al, 2009). In accordance with their embryonic ontology, DPSCs also express cell surface marker CD271, also known as low-affinity nerve growth factor (NGF) receptor, NGF receptor, or p75NTR (neurotrophin receptor) (Alvarez et al, 2015; Martens et al, 2012).

The presence of CD271 correlates with CD105 and neurogenic locus notch homolog protein 2 (Notch 2) expression, low proliferation, and high neurogenic differentiation potential (Alaidaroos et al, 2021; Alraies et al, 2017; Waddington et al, 2009). The lack of this central cell surface antigen marker on DPSCs is associated with high proliferation and colony-forming efficiency (Alaidaroos et al, 2021; Alraies et al, 2017). Altogether, these data suggest the importance of the appropriate identification and purification of DPSCs (Fig. 1).

Numerous studies have reported that hDPSCs are multipotent and able to differentiate into osteogenic (Liu et al, 2009), neurogenic (Arthur et al, 2008), adipogenic (Iohara et al, 2006), and chondrogenic lineages (Iohara et al, 2006). Multipotency closely relates to the biological roles of DPSCs as well as providing the stem cell pool for odontoblasts and for soft tissue regeneration in the dental pulp (Fawzy El-Sayed et al, 2013). Moreover, DPSCs can differentiate into neurogenic lineages that may correlate with their embryonic ontology (Arthur et al, 2008; Kiraly et al, 2011; Kiraly et al, 2009).

In comparison with other mesenchymal stromal stem cells, DPSCs have a higher neurogenic differentiation potential although the tissue heterogeneity leads to differences between the neurogenic DPSC-derived neurons. Using appropriate inductors, DPSCs can differentiate into functional neurons (Fig. 1) (Arthur et al, 2008; Kiraly et al, 2011; Kiraly et al, 2009).

## Neural Differentiation

Neurodegenerative diseases cause a progressive loss of functionally active, mature neurons. The replacement of damaged neurons as a therapeutic strategy has been extensively studied, and for regenerative medicine, it is crucial to find autologous, transplantable sources of neurons and glial cells (Barker et al, 2018; Bjorklund and Parmar, 2020; Volkman and Offen, 2017). Neural stem cells (NSCs) are stem cells found in the adult CNS and are able to differentiate into neurons, astrocytes, and oligodendrocytes. They reside in specific brain regions called neurogenic niches (Ming and Song, 2011). An adult human brain contains two neurogenic niches; (i) the subventricular zone of the lateral ventricles and (ii) the subgranular layer of the dentate gyrus. Although NSCs might be a source of transplantable neurons, their availability from the adult human brain is very limited.

Accessible stem cell sources can be found outside of the CNS (Ming and Song, 2011). Multipotent MSCs, including DPSCs, are able to differentiate into lineage-specific cell types (Dominici et al, 2006). This high differentiation potential correlates with great self-renewal capacity, easy accessibility, and high proliferation ability. Under standard culture conditions, growth factors and chemical inductors were used to differentiate hDPSCs into the neural lineage. During the last two decades, various differentiation strategies were developed to classify functionally active and subtype-specific neurons derived from hDPSCs. Table 1 summarizes the methodologies for neuronal differentiation of human DPSCs (hDPSCs). In general, neuronal induction and maturation of hDPSCs take between 1 and 3 weeks and were all isolated from non-inflamed human third molars.

**Table 1.** Summary of Neural Differentiation Methodologies Using Human Dental Pulp Stem Cells

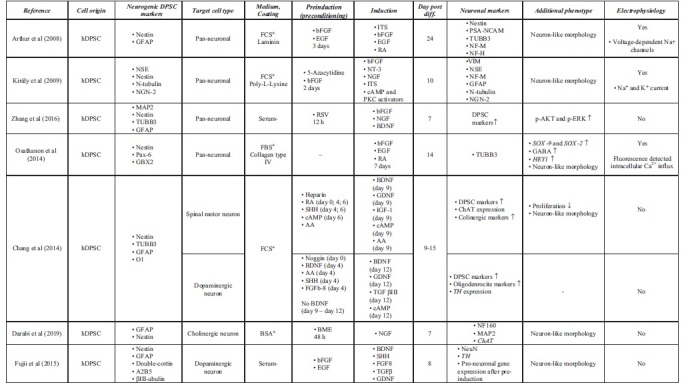 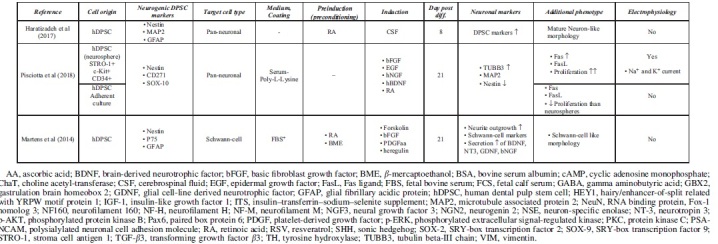

Growth factor-mediated approaches activate signal transduction pathways that may promote with neural lineage-specific gene expression (Karaoz et al, 2011). Chemical inductors such as forskolin or valproic acid significantly change the gene expression regulation of hDPSCs due to epigenetic modifications and signaling activation, therefore enhancing the conversion efficiency during neuronal differentiation (Heng et al, 2019). The separate usage of chemical inductors (butylated hydroxyanisole and β-mercaptoethanol) and growth factors, basic fibroblast growth factor (bFGF), epidermal growth factor (EGF), and retinoic acid (RA) was also studied, only resulting in a neuronal-like morphology on the differentiated DPSCs in case of growth factors (Table 1). The usage of chemical inductors, without growth factors, showed false-positive neural differentiation. Interestingly, gamma aminobutyric acid receptor expression was present after the growth factor-mediated differentiation. Functional activation of Notch signaling pathway was also observed in growth factor differentiated DPSCs (Osathanon et al, 2014).

Using chemical inductors and growth factors simultaneously, pan-neuronal and subtype-specific neuronal cells were differentiated, presented in detail in Table 1. Priming stimulation of the FGF/FGFR signaling pathway initiates cell cycle progression and proliferation through activating mitogen activated protein kinase/extracellular signal-regulated kinase (ERK) signaling in hDPSCs (Chang et al, 2017). Several studies reported that FGF/FGFR signaling also promotes neural differentiation in hDPSCs. In the presence of bFGF, increased hDPSC neurosphere size, neural morphology, and neurogenic marker upregulation were observed (Gonmanee et al, 2021; Li et al, 2019a; Nagashima et al, 2017; Osathanon et al, 2011). FGF stimulation supplemented with RA and EGF could not differentiate hDPSCs into subtype-specific neurons, but they were able to induce crucial morphological changes, which led to the functional activation of differentiated neurons by demonstrating their ability to respond directly to their surrounding environment (Arthur et al, 2008).

The presence and functional role of neurotrophic factors such as NGF are well reported from the dental pulp *in vivo*. NGF is a key regulator of the development of neuronal and non-neuronal cells. Low- and high-affinity NGF receptors, p75, and Tropomyosin receptor kinase (TrkA) respectively, were described at the cell surface of hDPSCs. In injured teeth, NGF and TrkA expression is upregulated, which shows that NGF signaling is strongly linked to the pathological and regenerative process of hDPSCs (Mitsiadis et al, 2017; Pisciotta et al, 2020). Following pretreatment with the demethylating agent 5-azacytidine and bFGF, activation of NGF and neurotrophin 3 (NT-3) signaling transduction pathway differentiated hDPSCs to functionally active, neuron-like cells in the presence of cyclic adenosine monophosphate (cAMP) and protein kinase C activators, such as IMBX (3-isobutyl-1-metilxanthin), forskolin, and tissue plasminogen activator (TPA) (Kiraly et al, 2009).

In the presence of RSV, NGF and bFGF promote the expression of neuronal markers (*Nestin*, *TUBB3*, *MAP2*) and induce neural differentiation through the ERK and protein kinase B signaling pathways (Zhang et al, 2017). Another neurotrophic factor, brain derived neurotrophic factor (BDNF), also plays a pivotal role in the neural commitment of DPSCs (Zhang et al, 2018). Luzuriaga et al (2019b) reported that BDNF and NT-3 are responsible for the differentiation of hDPSCs into NSCs *in vitro*.

Several studies investigated DPSC differentiation into subtype-specific neurons and glial cells including Schwann cells (Martens et al, 2014). Pre-exposure to bFGF and EGF and activation of MAPK/ERK signaling pathways, at the induction phase, appropriate neurotrophins from the embryonic development of spinal motor neurons or dopaminergic neurons are able to differentiate premature hDPSC-derived neurons to subtype-specific derivatives (Gonmanee et al, 2018; Vescovi et al, 1993). It has been reported that growth factors such as BDNF, glial cell-line derived neurotrophic factor (GDNF), NT-3, and NGF contribute to differentiate dopaminergic neurons *in vitro* (Fujii et al, 2015; Trzaska et al, 2009). In comparison with bone-marrow derived MSCs (BM-MSCs), neural-differentiated hDPSCs yield better cells in terms of neural-specific gene expression including the dopaminergic marker tyrosine hydroxylase (*TH*) (Singh et al, 2017).

Spinal motor neurons are localized at the ventral part of the spinal cord and during their embryonic development, several ventralization agents are contributing to their morphological fate determination, including sonic hedgehog (SHH) (Ravanelli and Appel, 2015; Yang et al, 2019). Using SHH in hDPSCs for the preinduction phase with their synergistic chemical inductor RA has promoted spinal motor neuron-like morphology and gene expression after being cultured with BDNF, GDNF, insulin-like growth factor 1 (IGF-1), and cAMP activators (Chang et al, 2014; Hua et al, 2021). Darabi et al (2019) also studied and presented a cholinergic differentiation of hDPSCs.

To maximize neuronal maturity and avoid undesired immune reactivity of transplanted DPSCs *in vivo*, the usage of xenogeneic serum elements is discouraged, although the growth rates of DPSCs are usually lower in serum-free environments than in the FBS-containing media (Gregory et al, 2006; Luo et al, 2018b; Luzuriaga et al, 2019a; Solis-Castro et al, 2020). Several protocols have been published to optimize the culture conditions of serum-free differentiated hDPSCs (Madanagopal et al, 2020; Sonoda et al, 2022; Zhang et al, 2017). Serum-free approaches are also applied to culture hDPSCs in spheroids, which may act as microniches providing a better culture environment to differentiate DPSCs (Bonnamain et al, 2013; Gonmanee et al, 2021). In comparison with adherent culture conditions, hDPSC spheroids show significant differences in neural gene expression, maturity, and proliferation potential (Gervois et al, 2015; Luzuriaga et al, 2019a; Xiao and Tsutsui, 2013).

In spheroid culture conditions, the expression of neural crest-derived genes (*Nestin*, *CD2171*, *SOX-10*) is notably higher than in adherent cultures, suggesting that spheres are favorable for the stemness and neuro-ectomesenchymal properties. In this process, insulin-like growth factor binding protein 5 (IGFBP5) plays a pivotal role by inducing the overexpression of neural cell adhesion molecule (NCAM), a key regulator of axon growth that promotes cell adhesion and migration. Under IGFBP5 stimulation, the number of Nestin- and TUBB3-positive neurospheres also increased (Chernyshova et al, 2011; Li et al, 2019b). It is noteworthy that differences between progenitor and mature neural gene expression were also described in spheres in comparison with adherent culture-derived hDPSCs. The relative expression of *TUBB3* and *MAP2*, which are both markers of neural lineage commitment, is higher at the same passage numbers than in adherent cultured hDPSCs.

In addition, differences in proliferation ability are also observed between adherent and spheroid conditions (Pisciotta et al, 2018). Notwithstanding, the expansion of hDPSCs cultivated on microspheres is also a promising direction for future studies (Foldes et al, 2021).

Interestingly, the cerebrospinal fluid (CSF) also provided a favorable microenvironment for differentiating hDPSCs into neurogenic lineages (Goudarzi et al, 2020). As the CSF is in direct contact with NSCs in the CNS, it contains neurotrophic and growth factors with other nutrients and cytokines, which are able to induce neural differentiation of hDPSCs with the simultaneous usage of RA. In addition, these RA/CSF-treated neurons passed the preneuronal stage and differentiated into mature neurons in induction culture due to the simultaneous activation of multiple molecular signaling transduction cascades. This condition is similar to the *in vivo* proceedings during embryonic development (Haratizadeh et al, 2017).

In summary, spindle-shaped hDPSCs can efficiently be differentiated into neuronal-like cells with neurite outgrowth and bulbous soma that express several neuronal markers (*e.g.*, NSE, NCAM, TUBB3, MAP2, NeuN). Electrophysiological analyses confirmed the functional presence of voltage-gated sodium and potassium channels involved in action potentials in hDPSC-derived neurons (Arimura et al, 2021; Arthur et al, 2008; Kiraly et al, 2009; Li et al, 2019a). Arimure et al (2021) found the presence of transient receptor potential (TRP) channels in the differentiated hDPSCs, by also examining the role of TRP channels during intracellular Ca^2+^ response. Ullah et al (2016) examined the functional maturity of differentiated hDPSCs and found a subpopulation-dependent electrophysiological maturation of hDPSC-derived neuronal cells. These data outline the importance of dental pulp tissue heterogeneity and how this affects the neurogenic differentiation of DPSCs.

## Discussion

Functionally active, mature neurons are anticipated to restore neural cells after injury or progressive loss of neurons in the CNS. To obtain hDPSC-derived neurons as an alternative cell source in regenerative medicine, various differentiation methodologies have been described. Cytoskeleton-associated proteins such as TUBB3, Nestin, GFAP, and MAP2 have been widely used in DPSC-based studies as neuronal markers (Govindasamy et al, 2010). It is important to emphasize that even these commonly used markers show a temporal expression profile during *in vivo* maturation of neurons. Undifferentiated hDPSCs, due to their neural crest origin, express both immature and mature neural markers (Rafiee et al, 2020). After neural differentiation, the change of relative marker expression levels is apparent, although there is no commonly accepted gold standard in the field so far as to which protocol leads to the optimal decrease of immature and increase of mature neural markers.

Another key aspect in cell therapy is the effective transplantation of differentiated hDPSCs to the target area *in vivo*. Scaffolds are biomaterials intended to cause desirable cellular interactions and support the three-dimensional formation of *in vitro* seeded cells (Henriques et al, 2019). hDPSCs conditioned with growth factors were able to grow and differentiate into neurons in highly porous chitosan scaffolds. *In vivo* transplantation of hDPSCs/chitosan scaffolds may provide a more physiologic microenvironment during attachment for transplanted hDPSCs (Zhang et al, 2016). *In vivo* injection of hDPSCs improved behavioral dysfunctions and survived several weeks in a neonatal hypoxic–ischemic brain damaged rat model (Fang et al, 2013). Combining hDPSCs with scaffold materials increased the expression of several neurotrophins such as BDNF, NT-3, or NGF, all of which are reported to be important for an enhanced tissue regeneration.

Synthetic and natural polymers such as heparin-poloxamer, poly-lactide-co-glycolide, and even chitosan polysaccharide promoted peripheral nerve regeneration in murine models (Luo et al, 2018a; Sasaki et al, 2011). Enhancing the neural maturity, functional activity, neurotrophin secretion, and transplantation efficiency of hDPSC-derived neurons into the host organism requires more research efforts to maximize the regenerative potential of hDPSCs. In line with this, scaffolds may serve as a promising solution for transplantation and integrating cells into the host organism.

Similar to other tissues, the dental pulp represents a heterogenous source of expanded cells, including a variable proliferation rate and cellular senescence. Alraies et al (2017) reported that there is a significant variability in the proliferative potential and cellular senescence between different patient-derived hDPSCs within a similar age range, and even identified inherent differences between hDPSC subpopulations derived from the same patient. Proliferative and regenerative heterogeneity is closely related to the replicative senescence (telomere length) and, interestingly, to the expression of the low-affinity NGF receptor, CD271. CD271^+^ cells showed a lower proliferation rate and a higher neurogenic potential suggesting that the presence of this specific neural crest marker was related to neuronal cell fate lineage restriction of hDPSCs (Alraies et al, 2017; Kok et al, 2022).

Selective screening for neurogenic niches of DPSCs within these subpopulations or the transplantation of nondifferentiated CD271^+^ cells for regenerative issues may lead to better results in the long term in DPSC-derived therapies. Overall, these data suggest the importance of population selection and selective screening from dental pulp tissue for *in vitro* expansion, aiding for more effective hDPSC-based therapies for clinical application.

During the last two decades, transcription-factor-mediated neuronal reprogramming approaches were developed. hDPSCs might be a promising cell source for pluripotent phase-mediated and direct reprogramming strategies (Tamaoki et al, 2010; Yan et al, 2010). Using fibroblasts as a cell source, the combinatorial expression of neural lineage-specific transcription factors led to directly converted neurons *in vitro* (Vierbuchen et al, 2010). These cells preserve donor age-dependent epigenetic and transcriptomic signatures and are able to model human aging in several late-stage neurodegenerative diseases such as Alzheimer's disease, Huntington's disease, and Parkinson's disease (Drouin-Ouellet et al, 2017a, Drouin-Ouellet et al, 2017b; Legault et al, 2022; Mertens et al, 2015; Pircs et al, 2022). In the case of DPSCs, transcription factor-mediated conversions were used to induce overexpression of POU domain class 5, transcription factor 1 (*OCT-4*), and *SOX-2*.

However, studies focused on the forced overexpression of specific neural lineage commitment transcription factors such as achaete-scute family BHLH transcription factor 1 (*ASCL1*), POU domain class 3 transcription factor 2 (*BRN2*), and myelin transcription factor 1 like (*MYT1L*) are not described yet (Liu et al, 2015a, Liu et al, 2015b).

Cellular heterogeneity is an important property of the dental pulp cell niche. Although individual hDPSC subpopulations share certain similarities, heterogeneity is a crucial factor considering the therapeutic benefits of DPSC usage (Kok et al, 2022). The previously mentioned cell marker CD271 certainly set an example for this phenomenon. High cellular proliferating ability is crucial in regenerative applications but does not simultaneously mean high neurogenic potential. In comparison with other transcription factor-mediated approaches or growth factor-mediated differentiation strategies, one of the main advantages of direct reprogramming is that transdifferentiated cells represent the whole cellular diversity of the donor tissue (Mertens et al, 2016). We propose that direct neuronal reprogramming using hDPSCs would be beneficial when cellular diversity is preferred while preserving the genetic and epigenetic identity of the donor. In addition, directly reprogrammed cells preserve the age-related signatures of donor cells (Drouin-Ouellet et al, 2017b; Mertens et al, 2016; Pircs et al, 2022).

Since hDPSCs can be harvested from extracted third molars, these cells can be easily accessed without ethical issues to obtain neuronal cells from young patients used for further studies (Yamada et al, 2019). Therefore, directly converted hDPSCs could provide an excellent cellular source for future studies focusing on neuronal aging and neuronal rejuvenation by using cells from donors of all ages. We postulate that transcription factor-mediated approaches with simultaneous usage of growth factor-mediated differentiation protocols may allow an increased neural maturity and differentiation efficiency. In summary, hDPSC-derived neurons can potentially overcome various limitations of stem cell differentiation by providing an efficient, easily accessible cellular source for neural cell restoration. This can greatly induce the development of novel regenerative therapeutic strategies in the future.
